# A highly invasive human glioblastoma pre-clinical model for testing therapeutics

**DOI:** 10.1186/1479-5876-6-77

**Published:** 2008-12-03

**Authors:** Qian Xie, Ryan Thompson, Kim Hardy, Lisa DeCamp, Bree Berghuis, Robert Sigler, Beatrice Knudsen, Sandra Cottingham, Ping Zhao, Karl Dykema, Brian Cao, James Resau, Rick Hay, George F Vande Woude

**Affiliations:** 1 Laboratory of Molecular Oncology, Van Andel Research Institute, 333 Bostwick Avenue NE, Grand Rapids, MI 49503, USA; 2 Laboratory of Noninvasive Imaging and Radiation Biology, Van Andel Research Institute, 333 Bostwick Avenue NE, Grand Rapids, MI 49503, USA; 3 Transgenic Core Program, Van Andel Research Institute, 333 Bostwick Avenue NE, Grand Rapids, MI 49503, USA; 4 Laboratory of Analytical, Cellular, and Molecular Microscopy, Van Andel Research Institute, 333 Bostwick Avenue NE, Grand Rapids, MI 49503, USA; 5 Program in Cancer Biology, Fred Hutchinson Cancer Research Center, Division of Public Health Sciences, 1100, Fairview Avenue North, Seattle, WA 98109, USA; 6 Department of Neuropathology, Spectrum Health Hospitals, 100 Michigan Street NE, Grand Rapids, MI 49503, USA; 7 Laboratory of Antibody Technology, Van Andel Research Institute, 333 Bostwick Avenue NE, Grand Rapids, MI 49503, USA; 8 Laboratory of Bioinformatics, Van Andel Research Institute, 333 Bostwick Avenue NE, Grand Rapids, MI 49503, USA

## Abstract

Animal models greatly facilitate understanding of cancer and importantly, serve pre-clinically for evaluating potential anti-cancer therapies. We developed an invasive orthotopic human glioblastoma multiforme (GBM) mouse model that enables real-time tumor ultrasound imaging and pre-clinical evaluation of anti-neoplastic drugs such as 17-(allylamino)-17-demethoxy geldanamycin (17AAG). Clinically, GBM metastasis rarely happen, but unexpectedly most human GBM tumor cell lines intrinsically possess metastatic potential. We used an experimental lung metastasis assay (ELM) to enrich for metastatic cells and three of four commonly used GBM lines were highly metastatic after repeated ELM selection (M2). These GBM-M2 lines grew more aggressively orthotopically and all showed dramatic multifold increases in IL6, IL8, MCP-1 and GM-CSF expression, cytokines and factors that are associated with GBM and poor prognosis. DBM2 cells, which were derived from the DBTRG-05MG cell line were used to test the efficacy of 17AAG for treatment of intracranial tumors. The DMB2 orthotopic xenografts form highly invasive tumors with areas of central necrosis, vascular hyperplasia and intracranial dissemination. In addition, the orthotopic tumors caused osteolysis and the skull opening correlated to the tumor size, permitting the use of real-time ultrasound imaging to evaluate antitumor drug activity. We show that 17AAG significantly inhibits DBM2 tumor growth with significant drug responses in subcutaneous, lung and orthotopic tumor locations. This model has multiple unique features for investigating the pathobiology of intracranial tumor growth and for monitoring systemic and intracranial responses to antitumor agents.

## Background

Human glioblastoma multiforme (GBM) is one of the most devastating cancers. Extensive tumor cell invasion occurs into normal brain parenchyma, making it virtually impossible to remove the tumor completely by surgery and inevitably causing recurrent disease [1]. There is therefore a compelling need for more reliable *in vivo *preclinical models for studying the disease and for testing new drugs and therapies. For GBM cell lines in common use, comparison of gene expression profiles from cell culture, subcutaneous xenografts, or intracranial xenografts can differ significantly within the same cell line; yet different GBM cell lines from orthotopic models exhibit similar gene profiling patterns [2]. Recent progress has been made in optimizing experimental models relevant to GBM. For example, glial progenitor cells can form invasive orthotopic glioblastoma tumors when driven by platelet-derived growth factor (PDGF) [3]. Lee *et al. *[4] established a culture system that allows tumor stem cells to grow in culture with basic fibroblast growth factor (bFGF) and epidermal growth factor (EGF) without serum, maintaining both genotype and phenotype similar to that of the primary tumor. Moreover, sorting of CD133-positive tumor stem cells from glioblastoma tumors yields highly angiogenic and aggressive orthotopic tumors in mice [5].

Significant progress also is being made in developing mouse models that are genetically engineered to develop GBM [6,7]. Another approach is to improve the orthotopic human xenograft GBM models. Most commonly used human GBM cell lines grow slowly as orthotopic xenografts or generate poorly invasive tumors in the mouse brain, bearing little resemblance to human GBM. Interestingly, although extracranial GBM metastases rarely happen [8-13], most human GBM tumor cell lines are metastatic from subcutaneous xenografts [14]. We used experimental lung metastasis (ELM) assays to enrich for metastatic cells. In this model, three of four commonly used GBM lines were highly metastatic, grew more aggressively in the brain and, after two cycles (M2), expressed highly elevated levels of Interleukin-6 (IL6), Interleukin-8 (IL8) and granulocyte macrophage colony-stimulating factor (GM-CSF), thereby resembling GBM in patients [15-18]. We further characterized one line, DBM2, which, when inoculated orthotopically, triggers vascular hyperplasia, and forms areas of central necrosis that are lined by a crowded aggregate of cancer cells. As DBM2 grows orthotopically it creates, in proportion to tumor growth, an opening in the calvarium that allows the use of imaging technologies for non-invasively evaluating and monitoring of therapeutic responses. Here we show that the HSP90 inhibitor 17-(allylamino)-17-demethoxy geldanamycin (17AAG) [19,20] significantly inhibits GBM DBM2 orthotopic growth.

## Methods

All experiments were performed as approved by the Institutional Animal Care and Use Committee (IACUC) and the Safety Committee of the Van Andel Research Institute.

### Cell culture

DBTRG-05MG, U87, and U118 are human glioma cell lines originally purchased from American Type Culture Collection (ATCC, Manassas, VA). DBM2 is a subclone of DBTRG-05MG derived through lung metastases after mouse tail vein injection as described below. U251 cells were provided by Dr. Han-mo Koo of the Van Andel Research Institute. All cells were grown in Dulbecco's Modified Eagle's Medium (DMEM) (GibcoTM, Invitrogen Corporation, Carlsbad, CA) supplemented with 10% fetal bovine serum (FBS) (Invitrogen Corporation) and penicillin and streptomycin (Invitrogen Corporation).

### Recovery of invasive GBM cells from lung metastasis

DBTRG-05MG, U251, U87 and U118 cells (10^6^) in 100 μl PBS were injected into nude mice via the tail vein. Individual mice were euthanized when moribund; the pulmonary lesions were collected at necropsy and transplanted subcutaneously into the flank of fresh host mice to propagate the tumors. To generate primary cultures, subcutaneous tumors were harvested at necropsy, washed in PBS, minced, and treated with 0.25% trypsin (Invitrogen Corporation) for 45 min. Released cells were collected at 1500 rpm and resuspended in complete DMEM containing 10% FBS. This procedure was repeated twice to obtain GBM-M2 cell lines. U251-M1 cells were harvested after 1 cycle of selection.

### Grading criteria of experimental metastasis

To compare the metastatic potential of GBM cell lines, 10^6 ^cells in 100 μl PBS were injected intravenously into nude mice. By time of necropsy, lungs were harvested and a scoring system was established as follows. If no visible lesions were observed in lungs or other organs, mice were scored as (-); if visible and/or hematoxylin and eosin (H&E)-stainable lung lesions were confined to ≤ 50% of the tissue section area, animals were scored as (+); if lesions in the lung exceeded 50% of tissue section area, animals were scored as (++); and if most of the lung was involved and a lesion was present in at least one other organ, animals were scored as (+++).

### Expression of cytokines and growth factors

To prepare GBM-conditioned media, 5 × 10^5 ^cells were seeded into 10-cm dishes and grown to 80% confluency. Cells were washed with PBS twice, and complete medium was replaced with DMEM lacking serum. After culture for an additional 24 hrs medium was collected and spun at 13,000 × rpm for 5 min (Sorvall RT7 Plus) and the supernatant fraction was collected and stored at -80C for *Multi-Analyte Profile (MAP) testing *(Rules-Based Medicine, Austin, TX). To do the data analysis, the concentration levels of cytokines and growth factors from each cell line was normalized based on cell numbers. The fold change in expression of 89 cytokines and proteins are determined by comparing expression levels of GBM-M2 sub-lines to their parental DBTRG-05MG, U87 and U251 cell lines. R version 2.6.1 was used to generate the heat-map of the expression level fold change.

### Intracranial injection

Immunocompromised [athymic nude (nu/nu)] mice at about six weeks of age were used for intracerebral injections. Mice were anesthetized using isoflurane gas anesthesia (~2%) and placed into the ear bars of a stereotaxic frame. A burr hole was created through the skull 2 mm posterior to the bregma, and 5 × 10^5 ^cells in 5 μl PBS were injected into the brain at 3 mm depth.

### Immunohistochemistry staining of GBM orthotopic tumors

Tumor tissues were harvested, fixed with formalin, and embedded in paraffin. Paraffin blocks were sectioned to perform H&E and immunohistochemistry (IHC) staining for microscopic evaluation. IHC was performed using the Discovery XT Staining Module (Ventana Medical Systems, Inc., Tucson, Arizona). Briefly, deparaffinized sections were incubated in Tris/Borate/EDTA, pH 8 at 95°C for 8 minutes and at 100°C for 36 minutes for antigen retrieval. For Met staining, slides were then incubated with primary antibodies MET4, a mouse monoclonal antibody (mAb) against the extracellular domain of human MET [21] at 1:250 dilution (8 μg/ml), anti-uPAR (R&D, Minneapolis, MN) at 1:200, and anti-CD31 (Neomarkers, Fremont, CA) at 1:200 for 60 minutes. The slides were then incubated with a universal secondary antibody, which is an anti-mouse and rabbit cocktail (Ventana Medical Systems, Inc.) for 30 minutes followed by diaminobenzidine (DAB) staining (Ventana Medical Systems, Inc.).

### Treatment of DBM2 mouse tumor models with 17AAG

17AAG was purchased from LC Laboratory (Woburn, MA). 17AAG was first dissolved in 100% DMSO and stored at -80°C and then freshly diluted with vehicle PBST (PBS with 0.05% Tween 80) just prior to injection [22]. For all tumor models, host mice (6-week old female nude mice) were given vehicle alone (control), 17AAG in vehicle at a daily dose of 20 mg/kg (single injection daily), or 60 mg/kg body weight (administered as two divided doses 6 hrs apart), all administered by intraperitoneal injection [22]. For drug testing in the GBM subcutaneous xenograft model, tumor volume (V_t_) was measured with manual calipers twice a week (V_t _= length × width × depth). Results are expressed as mean ± SE.

With the orthotopic GBM xenograft model, DBM2 cells were inoculated intracranially and tumor growth was monitored by serial high-resolution ultrasound as described in the supplementary figures [Additional Files 1 and 2]. Weekly measured tumor volume was normalized with the initial tumor size upon group to achieve the fold change of tumor volume. Result is expressed as mean ± SE. With lung metastasis model, 28 nude mice were divided into control (n = 8), 20 mg/kg (n = 10) and 60 mg/kg (n = 10) groups. Each mouse received a single intravenous tail vein injection of 10^6 ^DBM2 cells in 100 μl PBS. Treatment started the second day after the cells were injected and continued for 8 weeks, by which time most of the control mice were moribund. At necropsy, lungs were harvested and scored as described above; body weight and lung weight of each mouse were also recorded.

### Statistical analysis

Statistical analysis of 17AAG-treated DBM2 intracranial tumor growth was performed with a student's "t" test. Log-rank test was used to analyze survival time. Chi-square test was used for comparison of 17AAG treatments against DBM2 pulmonary metastases.

## Results

### GBM tumor cells have metastatic potential

Primary and metastatic brain tumors are often aggressive and exceedingly difficult to treat. Evaluating the efficacy of the novel targeted agents against brain tumors is problematic due to the inadequacy of relevant pre-clinical models. In contrast to metastasic cancers, GBM is highly invasive into the brain parenchyma and rarely fully resectable. Xenograft mouse models for human GBM inadequately recapitulate the human disease because of slow growth and invasion at the orthotopic location.

**Figure 1:**
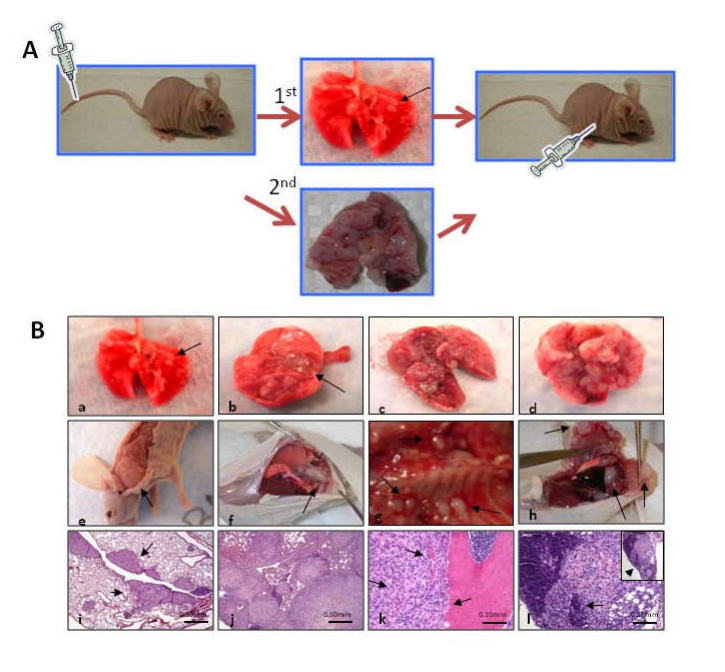
**In an experimental metastasis model, DBM2 cells produce tumors in various tissues**. (A) Clonal selection through experimental metastasis. The DBTRG-05MG cells were injected into the tail vein of athymic nude mice. Mice were sacrificed either when they became moribund (~12 weeks) or after 8 weeks. At necropsy, lung lesions were transplanted into nude mice subcutaneously. From these tumors, cells were harvested and injected into nude mice via tail vein. After the second cycle (M2) cells were expanded ex-vivo in culture. (B) DBTRG-05MG or DBM2 cells were injected via the tail vein into nude mice. After eight weeks mice inoculated with DBTRG-05MG cells had only a few pulmonary tumors (*a, b*). By contrast, lungs from mice bearing DBM2 cells were almost fully replaced with tumors (*c, d*), and metastatic foci were found in skeletal muscle (e), diaphragm (f), lymph nodes adjacent to the spinal cord (*g*) and in the chest cavity (*h*). H&E staining of formalin fixed sections from lungs of DBTRG-05MG cells (*i*) or DBM2 cells (*j*) eight weeks after tail vein injection. Invasion of DBM2 tumors into skeletal muscle (left 2 arrows) induces bone resorption (right arrow) (*k*) and replaces nearly the entire lymph node (arrow) (*l*, insert *at *low magnification).

We tested if we could enhance the growth and invasiveness of commonly used GBM lines by selecting metastatic cell populations from experimental lung metastasis (ELM). Clark *et al*. [23] used this approach to enrich for highly metastatic and invasive melanoma tumor cells. GBM extra-cranial metastases are rare [8,9,11-13], but surprisingly, most GBM cell lines tested have been shown to be metastatic from subcutaneous (SQ) tumor xenografts [14]. Here we show that three out of four GBM tumor lines are metastatic in ELM assays (Figure 1) and are more malignant when orthotopically grown (Table 1).

**Table 1:** Metastatic potential of commonly used GBM cell lines.

Cell line	Mouse NO (n)	(+)	(++)	(+++)
U118	5	0	0	0
				
U251	5	0	1	1
U251-M1	5	0	2	3
U251-M2	8	0	1	7
				
U87	5	0	0	2
U87-M1	7	0	3	4
U87-M2	10	0	3	7
				
DBTRG-05MG*	7	1	5	1
DBM2*	7	0	3	4

^§^To determine if invasive potential of GBM cells can be selected for *in vivo*, DBTRG 05MG, U251, U87 and U118 cells were subjected to experimental metastasis. 10^6 ^cells in 100 μl PBS were injected through the tail vein of nude mice. Mice were sacrificed when they were moribund, and lungs with tumors were scored and transplanted as described in Materials and Methods.

*For the comparison between DBTRG-05MG and DBM2, mice were sacrificed 8 weeks after tumor inoculation.

We started by injecting DBTRG-05MG cells into the tail vein of athymic nu/nu mice. DBTRG-05MG is a human glioma cell line that is highly invasive *in vitro *in response to hepatocyte growth factor (HGF), but grows poorly as SQ tumor xenografts [24,25]. Starting at 8 weeks after tail vein injection, we sacrificed mice individually and, when pulmonary tumor lesions were observed, we collected the lesions and propagated them *in vivo *as SQ tumors followed by a second cycle of ELM selection (M2). These cells, DBM2, were highly invasive and metastatic in ELM assays (Figure 1A, B). Tail vein injection of DBM2 cells produced extensive tumors almost replacing the lungs (Figure 1B, c–d, Table 1) compared to parental DBTRG-05MG cells, which only formed occasional and organ confined lung tumors (Figure 1B, a–b). DBM2 cells also formed extensive metastases in skeletal muscles (Figure 1B, e) diaphragm (Figure 1B, f), lymph nodes along the spine (Figure 1B, g), and in the chest cavity (Figure 1B, h). DBM2 cancer cells invaded skeletal muscle (Figure 1B, k left 2 arrows) and caused an osteolytic bone reaction consistent with the skull-erosion phenotype described below. DBM2 cells also grow more rapidly *in vitro *compared to parental DBTRG-05MG [Additional File 3] and especially *in vivo *as a xenograft, even compared to the GBM U251 line [Additional File 3][25].

We questioned whether more metastatic tumor cell populations can be selected by ELM from other commonly used GBM cell lines (U87, U251, U118): We were successful in selecting U87-M2 and U251-M2 cell lines after two ELM cycles. Both lines not only grew more rapidly, but as with DBM2, they showed extensive metastasis to lungs and other organs (Table 1). A comparison of tumor growth of U87 to U87-M2 either orthotopically or by ELM assay showed enhanced aggressive biological behavior of U87-M2 in both assays [Additional File 3]. When tested, all three GBM-M2 ELM lines showed significant growth enhancement in ELM, SQ or orthotopic xenograft mouse models (Table 1). By contrast, U118 GBM cells, which grow well as a SQ xenograft, did not form lung tumors in the ELM assay. Interestingly, when inoculated orthotopically, none of the GBM-M2 lines formed extracranial metastases. Why the metastatic potential of these intercranial tumors is not realized is curious, since these cancers are highly vascularized [Additional File 1;B,b], elicit marked angiogenesis (Figure 3C, e–f), and even display tumor cells in the tumor-associated vasculature (Figure 3C, d).

**Figure 2:**
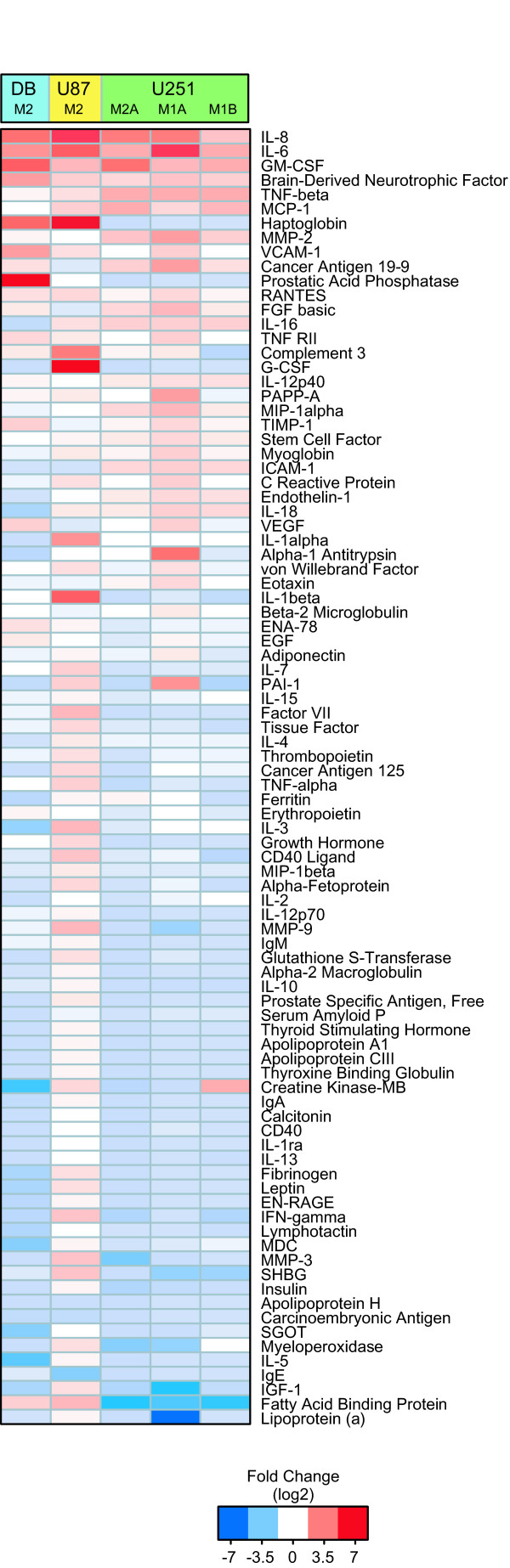
**Elevated cytokines and growth factors in GBM-M2 cells**. Identification of cytokines and growth factors in common in the 24 hr conditioned medium for all three GBM-M2 tumor lines and the fold increases in their expression compared to the parental GBM cells. Heat map shows fold differences based upon the of expression ratios of 89 cytokines and proteins between parental and GBM-M2 lines determined as described in the materials and methods section. The fold change in protein expression level is indicated by color. GM-CSF, IL-6, IL-8 and BDNF were found highly elevated in all three GBM-M2 lines (fold changes are summarized in the supplementary table [Additional File 4]).

**Figure 3:**
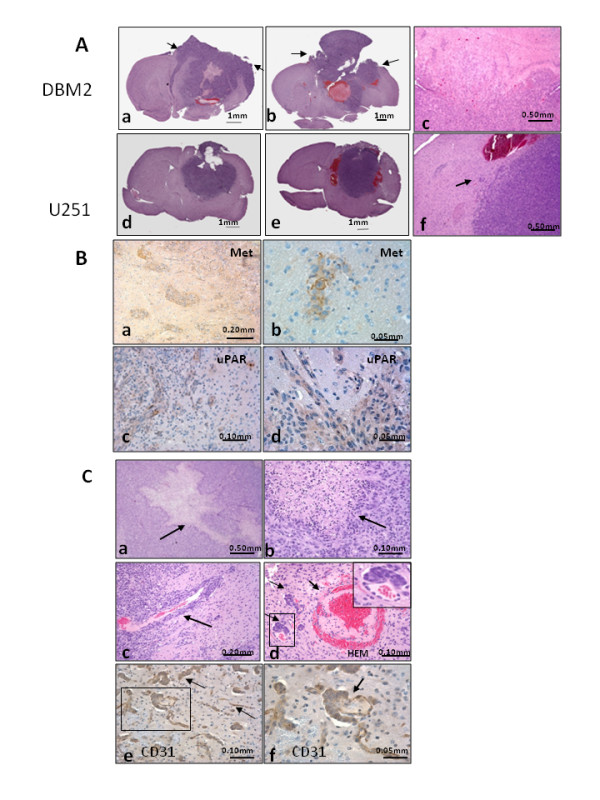
**Invasive growth and GBM properties of orthotopic DBM2 intracranial tumors**. (A) Orthotopic DBM2 tumors exhibit extensive infiltration into the mouse brain parenchyma (a, b). The arrows point to areas of cranial erosion. (c) Higher magnification of DBM2 tumor demonstrating extensive infiltration into the brain parenchyma. Compared to DBM2, U251 tumors form a sharper cranial margin (d, e) and are less invasive (f). (B) Met (a, b) and uPAR (c, d) expression in invasive DMB2 orthotopic tumors. (C) H&E staining of formalin fixed DBM2 tumors shows central necrosis with the crowding of cancer cells lining the necrotic area (a, b arrows). Vascular invasion of DBM2 tumors along the perivascular space (arrow) and in vessels in the surrounding brain (c) with tumor-thrombus formation (d). Higher magnification showing a glomeruloid body-like structure (d, insert). CD31 staining highlights vascular proliferation (e). Enlargement of (e) showing glomeruloid body-like structure with multiple layers of endothelial cells is stained by CD31 antibody (f).

### Elevated expression levels of cytokines and growth factors in GBM-M2 cells

The expression of a number of factors and interleukins is increased in patient GBM and is associated with glioma stage and aggressive tumor behavior [15-18]. Of note are pro-angiogenic cytokines and interleukins that are responsible for the vascular proliferation, a hallmark of GBM. We assayed 24 hr conditioned medium from the three GBM-M2 cell lines including U251-M1A and U251-M1B compared to their parental lines on a platform that queries expression of 89 proteins (Multi-Analyte Profile; Rules-Based Medicine, Austin, TX) . Figure 2 shows a heat map with fold changes described in the supplementary table [Additional File 4], revealing four cytokines and growth factors in all three GBM-M2 lines, GM-CSF, IL-6, BDNF, and IL-8 that were highly elevated in GBM-M2 cells (DBM2, U87-M2 and U251-M2) compared to their parental cell lines (DBTRG-05MG, U87 and U251). In addition, GM-CSF, IL-6 and IL-8 are all reported to be associated with poor prognosis in patient GBM [16,18]. In addition, monocyte chemotactic protein-1 (MCP-1), which is elevated in patients with GBM [26], is also highly elevated in U87 and U251 sub-lines. It is striking that GBM-M2 ELM selection of three separate cell lines markedly enhanced the expression of the same interleukins and cytokines that are of prognostic significance in GBM tumors. These results encouraged us to analyze the growth and histopathologic characteristics of this animal model for intracranial tumor growth.

### DBM2 orthotopic tumors are highly invasive in mouse brain and exhibit features associated with malignant GBM

Metastatic DBM2 cells grow orthotopically in mouse brain with a diffuse tumor boundary (Figure 3A, a–c) and finger-like protrusions (Figure 3A, c) indicative of infiltrative growth. Insufficient intracranial growth of parental DBTRG-05MG cells led to compare DBM2 intracranial growth with the orthotopic growth of parental U251 xenograft tumors. In contrast to DBM2 tumors, U251 tumors maintained a distinct border with the brain parenchyma with little localized invasion (Figure 3A, d–f). Analysis of tissue sections from DBM2 tumors for human c-MET and uPAR expression pinpointed the location of invasive glioblastoma cells in the brain parenchyma and at the same time examined an important mechanism for cellular invasion (Figure 3B). c-MET oncoprotein signaling promotes the activation of urokinase and its receptor (uPAR) [27] and both are associated with GBM invasion in patient tumors [24,27-29]. Adjacent to the main tumor xenograft, we observed human c-MET and uPAR staining of cells invading the normal brain parenchyma (Figure 3B) showing that DBM2 cells are highly invasive.

Certain pathological features are associated with aggressive behavior of many cancer types, including GBM [15,30]. DBM2 orthotopic tumors show many of these features. They are markedly pleomorphic and possess regions of central necrosis lined by a row of crowded tumor cells (Figure 3Ca, b arrows). Further, the orthotopic tumors exhibit extensive vascular hyperplasia (Figure 3Ce), vascular invasion (Figure 3Cd) as well as invasion of vessel walls (Figure 3Cc arrow), thrombus formation (Figure 3Cd). Glomeruloid body-like abnormal vasculature formation was observed upon staining with CD31 antibody (Figure 3Cf). Together, the invasive and aggressive growth behavior and cytokine profile of ELM selected xenografts strongly resemble human disease and validate this animal model for testing of drugs for inhibition of intracranial tumor growth.

### Real-time imaging of DBM2 tumor growth and vascularity

As DBM2 orthotopic tumors grow, we observed that the opening created for tumor cell inoculation increases in size, allowing both intra and extracranial tumor growth [Additional File 1]. This opening allows high-resolution intravital imaging of DBM2 tumor growth [Additional File 1;B]. Ultrasound imaging revealed poorly distinct tumor margins, consistent with invasive growth. Further, ultrasound measurements demonstrated that the increase of tumor volume was accompanied by a proportional increase of the skull erosion at the DBM2 cell inoculation site [Additional File 2]. This was confirmed by CT technology (data not shown). We compared the dimensions of the skull erosion obtained by ultrasound [Additional File 1;A,c], the distance between the arrows) to measurements with conventional calipers [Additional File 1;A,d] at the time of necropsy and observed good correlation between the two approaches (γ = 0.87, n = 10). Beneath the skull erosion, tumor volume was determined from the ultrasound images [Additional File 2;C]. Moreover, we found a high correlation (γ = 0.95, *n *= 96), [Additional File 2;D] between tumor volume and the size of the skull opening measured by ultrasound. Thus, the skull opening provides a simple way to monitor tumor growth during therapeutic intervention.

We found that, with Doppler and contrast injection ultrasound, both the amount of blood flow and the direction of the flow in the orthotopic DBM2 tumor can easily be visualized. Under the Doppler mode [Additional File 1;B, a], we see strong energy signals that accumulate in the skin, indicating the existence of "macro" blood vessels with high blood flow in these tissues. However, the tumor mass is mostly dark, indicating that the tumor vasculature does not emit a Doppler signal. To enhance the visualizing of tumor blood vessels, we injected a contrast reagent through the tail vein before ultrasound measurement. Following injection, we saw a rich vascular network extending from the bone-tumor margins along the intracranial boundary of the tumor [Additional File 1;B,b]. Strikingly, almost all the tumor provided a contrast signal, indicating that the DBM2 orthotopic tumors have micro-blood vessels with a lower flow rate than abundant large, mature blood vessels. This makes the DBM2 intracranial glioblastoma model particularly useful as a preclinical model to evaluate novel therapeutic interventions against vascular flow and formation. Given the resemblance of this animal model to patient GBM we proceeded with the evaluation of the 17AAG for inhibition of intracranial tumor growth.

### 17AAG inhibition of DBM2 tumor growth and metastasis

**Figure 4:**
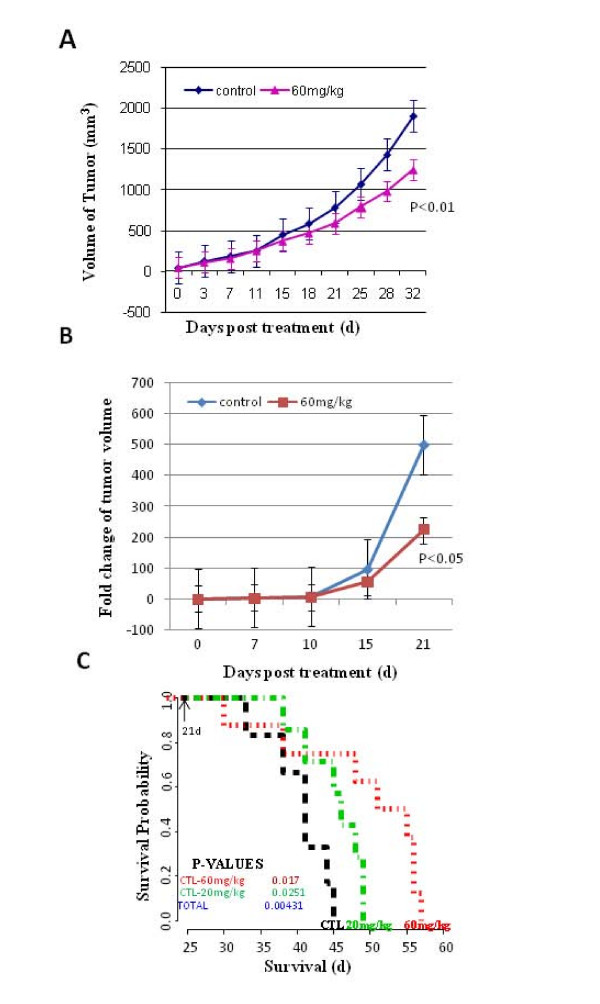
**17 AAG inhibition of DBM2 tumor growth**. (A) 17AAG at 60 mg/kg-d inhibits DBM2 subcutaneous tumor growth. DBM2 cells were inoculated into the flanks of nude mice at 5 × 10^5 ^cells in 100 ul PBS. After 2 weeks, mice with size-matched tumors (100 – 200 mm^3^) were assigned into control and treatment (60 mg/kg-d) groups (n = 19) and treatment started. Error bar represents for standard error. (B) 17AAG at 60 mg/kg-d inhibits DBM2 orthotopic tumor growth. DBM2 cells were inoculated intracranially into nude mice at 5 × 10^5 ^cells in 5 ul PBS. The tumor growth was monitored by Ultrasound. After 2 weeks, size-matched tumors were grouped into control and treatment groups (n = 10). Fold change of tumor volume = Weekly measured tumor size/Initial tumor size upon grouping. (C) The survival time of nude mice bearing orthotopic DBM2 tumor xenografts treated with 17AAG. DBM2 cells were inoculated intracranially of nude mice at 5 × 10^5 ^cells in 5 ul PBS. After 3 weeks, size-matched tumors were grouped into control (n = 6) and 2 treatment groups (20 mg/kg, 60 mg/kg, n = 8). The arrow points to the day treatment started after orthotopic tumor inoculation. Treatment was administered until individual mice became moribund according to IACUC guild-line and survival time was recorded.

17AAG is an HSP90 inhibitor that is in clinical phase I trials targeting different types of cancers, but its use has not been reported against glioblastoma [19,20,31]. With the SQ model, 17AAG at 60 mg/kg gave significant growth inhibition after 4 weeks of dosing (Figure 4A, P < 0.05 at day of 32). When the orthotopic model was used, however, results with the 60 mg/kg-day group growth rate was significantly lower than that of mice in the non-treated DBM2 control group (Figure 4B, P < 0.05 at day 21). Moreover, administration of 17AAG at 60 mg/kg-day significantly prolong the survival of mice bearing DBM2 intracranial tumors in dose-dependent manner (Figure 4C, p < 0.05).

We also tested if 17AAG can inhibit DBM2 ELM metastasis, for the purpose of determining whether the drug would inhibit this invasion dependent metastasis assay. Our results show that, at 60 mg/kg-day, 17AAG can significantly block DBM2 metastasis formation in lungs and other organs (Table 2, P < 0.05). Moreover, the harvested lungs from the 60 mg/kg-day group demonstrated significantly less tumor burden than those from the 20 mg/kg-day and control groups (Table 2, P < 0.05). We conclude that 17AAG inhibits intracranial DBM2 tumor growth at the same dose (60 mg/day) as tumor growth and metastasis formation in the SQ and ELM models. This strongly encourages testing of a novel application for 17AAG in patients with GBM.

**Table 2:** 17AAG inhibits the development of DBM2 pulmonary lesions.

				Lung grade		
				
Group	17AAG dose (mg/kg-d)	Body weight (g)	Lung weight (g)	**+**	**++**	**+++**
1 (*n *= 8)	Vehicle only	17.79 ± 1.88	0.477 ± 0.19	2 (25%)	3 (37.5%)	3 (37.5%)
2 (*n *= 10)	20	19.88 ± 1.68*	0.412 ± 0.17	3 (30%)	2 (20%)	5 (50%)
3 (*n *= 10^§^)	60	20.17 ± 0.89*	0.276 ± 0.11*	8 (80%)	2 (20%)	0

*Compared with group 1; Student's *t *test was used (p < 0.05)

§Compared with group 1; Chi-square was used for statistical analysis P < 0.05.

For drug testing in the lung metastasis model, 28 nude mice (6-week-old females) were divided into three groups: a control group (*n *= 8), and 17AAG groups treated with either 20 mg/kg (*n *= 10) or 60 mg/kg (*n *= 10). Each mouse received a single intravenous tail vein injection of 10^6 ^DBM2 cells in 100 μl PBS. Treatment started the second day after the cells were injected and continued for 8 weeks, by which time most of the control mice were moribund. At necropsy, lungs were harvested and scored; body weight and lung weight of each mouse were also recorded.

## Discussion

The limited number of preclinical models that recapitulate the invasive GBM tumor growth is a major hurdle to drug development. Subjecting human melanoma cells to ELM yielded highly metastatic cells with higher proliferative and invasive potential [23,32]. We applied this method to GBM cell lines for the purpose of improving their invasiveness in orthotopic models. The ELM assay has been used to select for metastatic cancer cells in a number of other cancer types [33-35], but has not been tested previously with GBM, most likely because of the notion that extracranial metastases of human GBM are clinically rare.

Here we show that GBM cell lines can be highly invasive after ELM selection, but they still are not metastatic when implanted in the brain. The lack of extracranial metastasis of the derivative GBM-M2 cell lines strongly suggests that rapid tumor growth or the unique CNS environment curtails the escape of tumor cells [14]. A previous study confirms the intrinsic metastatic nature of GBM tumor cells: GBM tumor cells were metastatic in spontaneous metastasis assays and no different than other types of cancer cells when tested in these assays [14]. Although stem cells isolated from primary tumor tissues [4,36] have not yet been tested for metastatic potential, the stem-cell like sub-populations from rat C6 glioma cells form neurospheres and like our GBM-M2 cells, are metastatic to lungs, as well as to other organs in nude mice upon intraperitoneal (i.p.) injection [37], again supporting that GBM tumor cells have intrinsic metastatic potential. Consistent with these reports we show that three of four commonly used GBM lines are highly metastatic in ELM assays (Table 1) and form metastasis in lungs and lymph nodes, similar to the destinations of some of the rare clinical GBM metastases in patients [8,9,11-13]. It is quite remarkable that GBM tumor cell lines, which came from primary tumors that have never grown as metastases and are selected to grow *in vitro *in tissue culture, have the capacity to be highly metastatic. This indicates that some aspect of GBM malignancy also satisfies the requirements for the metastatic process, or that the metastatic genotype is acquired early in tumor progression as has been proposed [38,39]. We have proposed that once cells acquire an invasive phenotype, they have the ability to acquire a proliferative phenotype again to become a metastatic colony [40].

The changes in cytokine and growth factor expression that occur after ELM GBM-M2 selection are similar to those that predict aggressive disease and poor patient outcome, demonstrating the similarity of cell lines to the scenario in patients. Interestingly, after ELM selection, all three GBM-M2 lines show highly elevated GM-CSF, IL-6, IL-8 and Brain-derived neurotrophic factor (BDNF) compared with parental cell lines (Figure 2, [Additional File 4]). Both GM-CSF and its receptor are absent in normal brain but expressed at high levels in glioma tissues [17]. *In vitro*, GM-CSF stimulates glioma cells to both proliferate and migrate [17]. IL-6 gene amplification in patients distinguishes GBM from low-level astrocytoma and is associated with poor prognosis [18]. In addition, IL-8 expression is highly associated with gliomagenesis and tumoral angiogenesis. Taken together, the co-elevation of these 3 cytokines appears to be an important indicator for GBM or poor prognosis. BDNF, a member of the neurotrophin family, plays an important role in neuronal development and survival [41]. Although a role for BDNF in GBM is not elucidated, its downstream signaling through Ras, ERK as well as PI3K pathways [42], would suggest it could play a role in GBM disease. Furthermore all of the GBM lines express high levels of MCP-1, also a marker of poor prognosis in patient gliomas [26]. All of these markers are consistent with the GBM nature of the GBM-M2 cells.

We chose to further develop DBM2 cells as an orthotopic model. DBM2 cells, when inoculated orthotopically, not only show significant invasive growth, but also central necrosis, extensive vascular hyperplasia, and glomeruloid body-like vasculature formation.  Brat et al. (2004, 2005) have reported the pathological features associated with poor diagnosis in GBM patients as well as the possible mechanisms. Necrosis is a hallmark of glioblastoma occurring in 60% of GBM patients while intravascular tumor-thrombus formation is found in over 90% of GBM cases. In addition, vascular hyperplasia is a characteristic of GBM and associated with poor prognosis [15,30,43]. As an explanation for their highly invasive nature, we show that DBM2 tumors not only express both c-Met and uPAR, the receptor of urokinase signaling pathway, but also strongly respond to HGF (data not shown) indicating that the c-Met signaling pathway may play an important role in the invasion of DBM2 orthotopic tumors into the brain parenchyma [24,27,40,44]. Brain tumors seldom invade the skull, but there are reports of GBM with skull-erosion phenotypes and metastases to other organs [45,46]. The exact mechanism of the osteolytic phenotype of DBM2 is unknown. It is possibly mediated through activation of bone-resorbing osteoclasts and may be facilitated by elevated IL-6 and IL-8 levels [47,48].

Real-time noninvasive imaging technologies permit longitudinal monitoring of tumor progression. Magnetic resonance imaging (MRI) is commonly used for human brain tumor imaging and is being refined in preclinical models [7]. Bioluminescence-based *in vivo *imaging systems are also used to rapidly measure tumor volume and evaluate drug efficacy in animal models [49]. Cranial window models have been developed in which part of the mouse skull is replaced with a cover glass so that the blood vessels can be observed microscopically [50]. Here, taking advantage of the osteolytic phenotype, we show high-resolution ultrasound can be used to monitor real-time, non-invasive imaging of brain tumor growth and vascularization. In addition, with Doppler and contrast injection ultrasound, directional blood flow can easily be visualized in the tumor.

We show that our xenograft model is versatile in that it can be used with SQ implantation for measuring tumor growth potential [25], with systemic injection for measuring invasive and metastatic growth potential in EML assays [51], or with orthotopic administration of tumor cells for measuring tumor growth in a macro- and micro-environment that recapitulates GBM in patients. Thus this model is particularly suitable for testing therapeutics. We chose here to test the drug, 17AAG, because of its diversity in targeting the destabilization of numerous oncoproteins [52]. 17AAG, a derivative of geldanamycin, an HSP90 inhibitor that has been in clinical trials in patients with advanced cancer [19,20]. It has not been considered for GBM treatment largely, we suspect, because of anticipated blood brain barrier interference with drug delivery. We show here that in all three tumor settings, 17AAG at 60 mg/kg, significantly inhibits tumor growth (Table 2, Figure 4). Thus 17AAG prevents SQ xenograft formation, the formation of metastatic lesions in ELM assays and importantly, at the same dose, inhibits DBM2 orthotopic tumor growth and prolongs animal survival time. It is certainly possible that the highly invasive GBM tumors compromise the BBB in our DBM2 orthotopic model leading to significant 17AAG anti-tumor activity. Studies with orthotopic GBM mouse models have shown that imaging reagents can leak from the intracranial tumors, indicating that the BBB is compromised [7] and anti-HGF mAbs, despite their large molecular size can inhibit orthotopic tumor growth in the brain [53,54]. Our results indicate that 17AAG may be used clinically to treat malignant GBM patients providing there is limited BBB interference with drug penetration.

In conclusion, we report that commonly used GBM cells have metastatic potential which can easily be selected in ELM assays. When implanted in the brain, the metastatic potential of GBM cells can be converted to a highly invasive phenotype. Importantly we show that 17AAG is an effective inhibitor of orthotopic tumor growth and that the response to treatment can be measured in real-time by ultrasound. We anticipate that this orthotopic model with high-resolution ultrasound technology will serve as a valuable tool in preclinical screening for drugs effective in targeting GBM.

## Competing interests

The authors declare that they have no competing interests.

## Authors' contributions

QX designed study, isolated and characterized cell lines, performed ultrasound imaging, performed data analysis and interpretation and prepared manuscript. RT performed animal experimentation. KH served as sonographer. LD performed ultrasound imaging and assisted with animal studies. BB performed immunohistochemistry, staining procedures and evaluation. RS reviewed pathological slides and provided interpretation. BK served as pathologist and assisted with preparation of manuscript. SC served as pathologist. PZ prepared Met4 antibody. KD performed statistical analysis. BC prepared Met4 antibody. JR performed histology and immunohistochemistry. RH performed ultrasound imaging. GVW developed the concept and designed study, interpreted data, prepared manuscript, and supervised study.

## Supplementary Material

Additional file 1 **DBM2 orthotropic tumor growth promotes cranial osteolysis**. The data provided demonstrate the rationale of using cranial osteolysis phenotype to perform ultrasound imaging. Click here for file

Additional file 2 **DBM2 orthotopic tumor growth promotes cranial osteolysis-continued**. Ultrasound imaging reveals that the cranial osteolysis generated by DBM2 orthotopic tumor growth results in an opening that is proportional to tumor size. Click here for file

Additional file 3 **GBM-M2 cells show enhanced malignancy in vitro and in vivo compared to GBM cells**. The data provided include the growth curves and survival time of GBM-M2 cells compared with the parental cell lines. Click here for file

Additional file 4 **Fold increases of cytokines and growth factors in GBM sub-lines**. The data provided represent the fold changes of cytokines and growth factors amongst all three GBM-M2 lines. Click here for file

Additional file 5 **Supplementary Materials & Methods**. The data provided represent the materials and methods used for Additional Files 1, 2, 3, 4 (this file is not cited in the paper; it is the Materials and Methods used for the supplementary figures). Click here for file

Additional file 6 **Supplementary Figure Legends**. This file contains the figure legends for supplementary Figures 1 and 2 (this file is not cited in the paper; it contains the supplementary figure legends). Click here for file
