# Supplementary figures and images for: A highly invasive human glioblastoma pre-clinical model for testing therapeutics
Source: J Transl Med. 2008 Dec 3;6:77. doi: 10.1186/1479-5876-6-77 (PMC2645376; doi:10.1186/1479-5876-6-77)

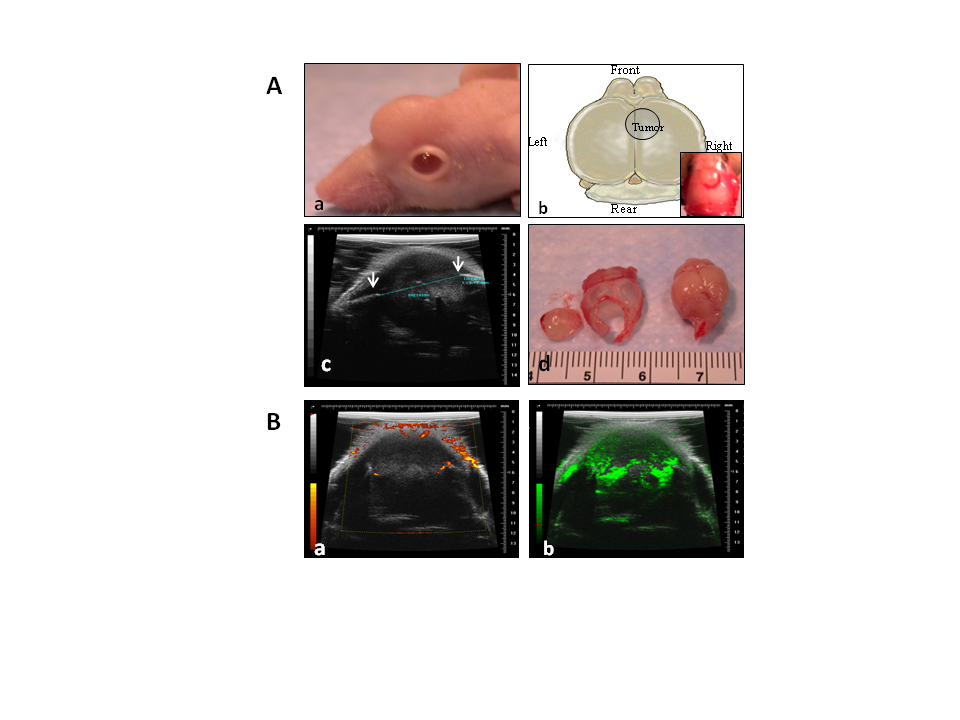

Supplement: Additional file 1 — DBM2 orthotropic tumor growth promotes cranial osteolysis. The data provided demonstrate the rationale of using cranial osteolysis phenotype to perform ultrasound imaging. [file 1479-5876-6-77-S1.tiff]

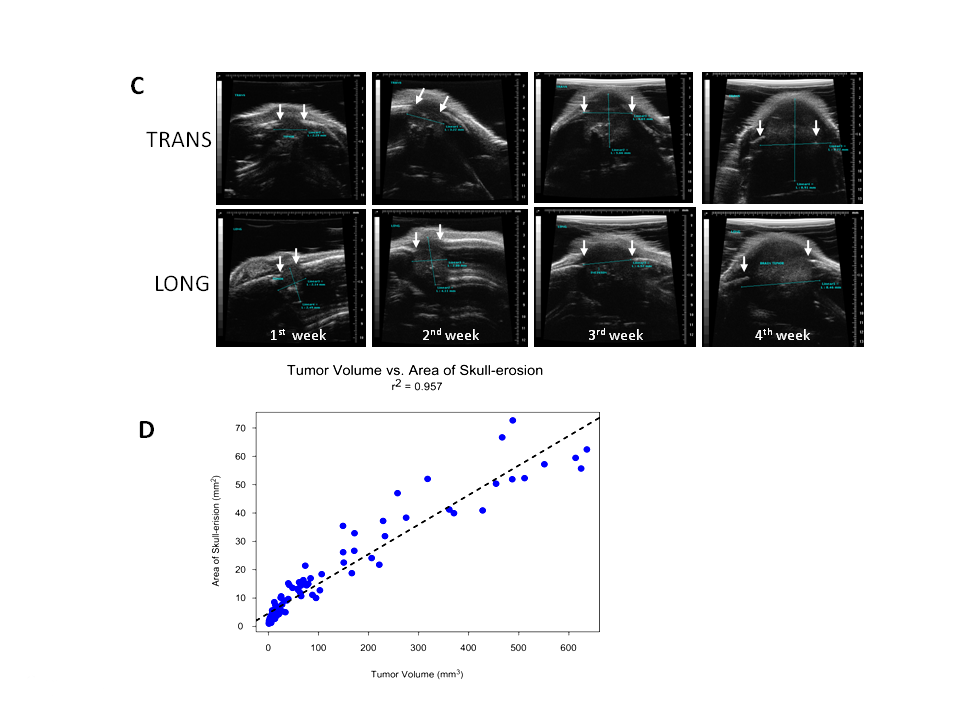

Supplement: Additional file 2 — DBM2 orthotopic tumor growth promotes cranial osteolysis-continued. Ultrasound imaging reveals that the cranial osteolysis generated by DBM2 orthotopic tumor growth results in an opening that is proportional to tumor size. [file 1479-5876-6-77-S2.tiff]

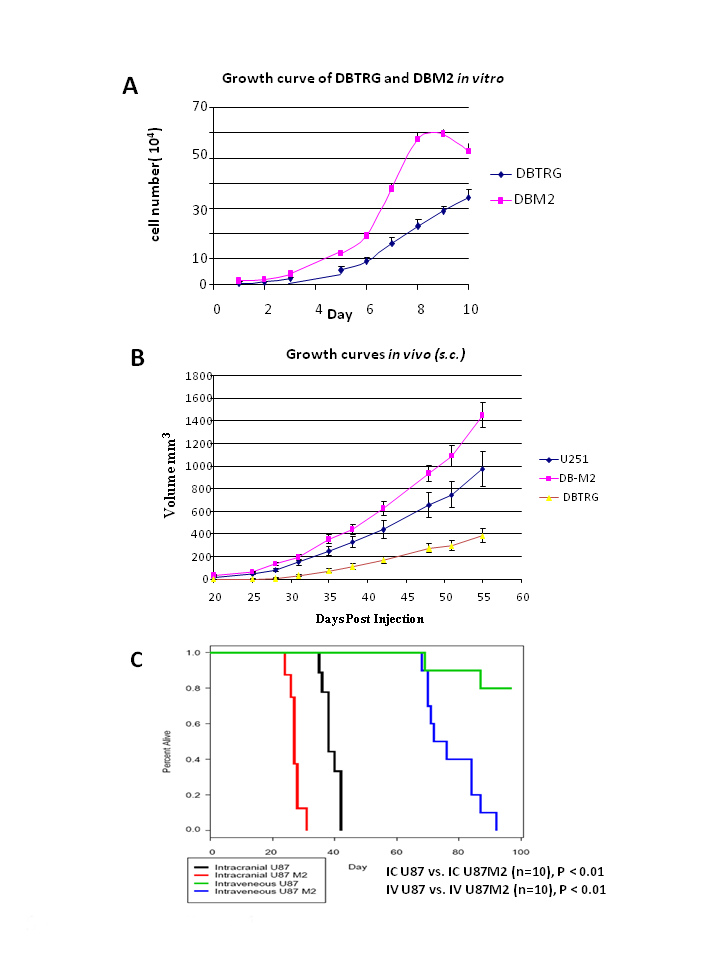

Supplement: Additional file 3 — GBM-M2 cells show enhanced malignancy in vitro and in vivo compared to GBM cells. The data provided include the growth curves and survival time of GBM-M2 cells compared with the parental cell lines. [file 1479-5876-6-77-S3.tiff]
