# Supplementary material for: A highly invasive human glioblastoma pre-clinical model for testing therapeutics
Source: J Transl Med. 2008 Dec 3;6:77. doi: 10.1186/1479-5876-6-77 (PMC2645376; doi:10.1186/1479-5876-6-77)
Supplement: Additional file 4 — Fold increases of cytokines and growth factors in GBM sub-lines. The data provided represent the fold changes of cytokines and growth factors amongst all three GBM-M2 lines. [file 1479-5876-6-77-S4.doc]

**Supplementary Table**

**Supplementary Table 1. Fold increases of cytokines and growth factors in GBM sub-lines.**

|  | **DBM2** | **U87M2** | **U251M2A** | **U251M1A** | **U251M1B** |
| --- | --- | --- | --- | --- | --- |
| IL-8 | 14.16 | 33.54 | 10.45 | 12.43 | 3.74 |
| IL-6 | 7.03 | 19.29 | 4.90 | 33.31 | 4.75 |
| GM-CSF | 18.82 | 4.08 | 13.42 | 4.09 | 5.06 |
| BDNF | 6.87 | 2.71 | 2.48 | 3.30 | 3.11 |
| TNF-beta | 1.26 | 1.94 | 5.17 | 5.55 | 4.98 |
| MCP-1 | 1.12 | 3.15 | 4.93 | 2.32 | 4.62 |

Fold changes of cytokines and growth factors in common amongst all three GBM-M2 lines. Six out of 89 proteins show the same increasing or decreasing trends and four, IL6, IL8, GM-CSF and MCP-1 have been associated with GBM and poor prognosis.
