# Supplementary material for: A highly invasive human glioblastoma pre-clinical model for testing therapeutics
Source: J Transl Med. 2008 Dec 3;6:77. doi: 10.1186/1479-5876-6-77 (PMC2645376; doi:10.1186/1479-5876-6-77)
Supplement: Additional file 5 — Supplementary Materials & Methods. The data provided represent the materials and methods used for Additional Files 1, 2, 3, 4 (this file is not cited in the paper; it is the Materials and Methods used for the supplementary figures). [file 1479-5876-6-77-S5.doc]

**Supplementary Materials and Methods**

**In vitro and in vivo Growth curves**

For *in vitro* assays, GBM cells were seeded in 24-well plates at 5000 cells per well and grown in DMEM with 10% FBS. Each day, cells from 3 wells were trypsinised and counted for total cell number for up to 10 days. For an *in vivo* study, 5x 10^5^ cells in 100 µl PBS were inoculated into nude mice subcutaneously. Tumor size was measured with calipers and tumor volume was determined [*V*(mm^3^) =length*width*depth] every other day.

**Survival time of intracranial and experimental metastasis assays.**

For intracranial models, U87 or U87M2 cells were inoculated into 10 nude mice brain at 5x10^5^ cells. For experimental lung metastasis assay, U87 or U87M2 cells were injected into 10 nude mice at 10^6^ cells via tail vein. Individual mouse was sacrificed when moribund according to the IACUC guide line and the days of survival was recorded.

**Ultrasound imaging for brain tumor parenchyma and vascularity measurements.**

Using the VisualSonics Vevo 770® High Resolution *In vivo* Micro-Imaging System, ultrasound images were obtained from intracranial human GBM xenografts raised in nude mice. Mice were anesthetized using Avertin (30mg/kg) and laid prone on the platform. The platform and ultrasound gel were continuously warmed to support mouse euthermia. For serial tumor measurements, high-resolution images (~30 micron spatial resolution) were acquired with the Vevo 770 system using the RMV 706 probe in B mode at a frequency of 40 MHz, and tumor dimensions were determined with system-included software. To evaluate tumor vascularity, for each host animal a baseline image sequence was first acquired using the RMV 706 probe in contrast mode at a frequency of 40 MHz, and then a dynamic sequence was acquired beginning just prior to contrast ultrasound microbubble injection, continuing for up to 60 seconds, or a total of 675 frames post injection. Vials of contrast ultrasound microbubbles (supplied as Vevo® MicroMarker™ Non-Targeted Contrast Agent Kit (VisualSonics, Inc., Toronto) were reconstituted into suspension with injectable saline according to the distributor’s recommendation. For each injection a bolus of 1x 10^8^ bubbles was administered in a volume of 50 μl via a 27 G hypodermic needle into a lateral tail vein using direct visualization. All imaging studies described herein were conducted by trained and qualified personnel in accordance with IACUC approval.
