# Supplementary material for: A highly invasive human glioblastoma pre-clinical model for testing therapeutics
Source: J Transl Med. 2008 Dec 3;6:77. doi: 10.1186/1479-5876-6-77 (PMC2645376; doi:10.1186/1479-5876-6-77)
Supplement: Additional file 6 — Supplementary Figure Legends. This file contains the figure legends for supplementary Figures 1 and 2 (this file is not cited in the paper; it contains the supplementary figure legends). [file 1479-5876-6-77-S6.docx]

**Supplementary Figure 1 [Additional Files 1 and 2]. DBM2 orthotopic tumor growth promotes cranial osteolysis, resulting in an opening that is proportional to tumor size.**

(A) Using Ultrasound to perform tumor growth measurement. (a and b) As DBM2 tumors grow, the opening in the calvarium increases at the site of inoculation allowing tumor growth above and below the cranium. A mouse is placed on a heated platform with the head in a dorsal position to the ultrasound probe; the tumor appears in the front-right quarter. A series of images was taken from both transverse (from front to rear) and longitudinal (from left to right) positions to record X-Y tumor axis specifications (inset picture shows where the DBM2 tumor erodes the skull). (*c*) The image showing the use of ultrasound to measure the dimension of skull-erosion (the distance between the arrows) (*d*) At the time of necropsy (eight weeks), the dimension of skull-erosion of was measured with calipers and compared to opening of the same mouse measured with ultrasound.

(B) The DBM2 intracranial tumors also permits high-resolution ultrasound images of blood flow in and near the tumor. (a) Power Doppler images show the highest blood flow velocity occurs in the scalp vessels and near the residual cranial edges. *(b)* By contrast, ultrasound images following tail vein injection of contrast microbubbles reveals the highest vascular density along the junction of the extracranial and intracranial segments of the growing tumor.

(C) The skull-erosion of DBM2 permits the real time measuring of the orthotopic tumor volume with ultrasound. Beginning two weeks after intracranial inoculation ultrasound measurements were performed weekly to measure tumor size. Through the area of skull-erosion (arrows), is detected with ultrasound and the tumor length, width or depth were recorded from either TRANS or LONG position. Tumor Volume was calculated as V = (length*width^2^) /2). With same mouse, the dimension of skull-erosion at both TRANS and LONG positions were also recorded (marked as the distance between the two arrows) and the area of skull-erosion was calculated as AO= (0.8*length*width).

(D) The tumor volume and skull opening determined from ultrasound measurement shows high correlation (γ^2^=0.96, n=96)

**Supplementary Figure 2 [Additional File 3]. GBM-M2 cells show enhanced malignancy *in vitro* and *in vivo* compared to GBM cells.**

(A) *In vitro* growth curve of DBTRG-05MG compared to DBM2 cells. (B) Subcutaneous tumor growth comparison *in vivo* of DBTRG-05MG, DBM2, and U251 cell xenografts. (C) Experimental lung metastasis selection enhances U87 malignancy. U87 and U87M2 cells were inoculated into nude mice via intracranial injection (IC, at 5X10^5^ cells) or tail vein injection (IV, at10^6^ cells). Moribund mice were sacrificed and survival time was recorded and compared. Upon necropsy, three U87M2 mice scored as “++” and seven mice as “+++” (Table 1).
